# Genomic selection using a realized genomic relationship matrix in a *Pinus taeda* L. cloned population

**DOI:** 10.1186/1753-6561-5-S7-P60

**Published:** 2011-09-13

**Authors:** Jaime Zapata-Valenzuela, Fikret Isik, Christian Maltecca, Jill Wegryzn, David Neale, Steven McKeand, Ross Whetten

**Affiliations:** 1 Cooperative Tree Improvement Program, Department of Forestry and Environmental Resources, North Carolina State University, Raleigh, NC, USA; 2 Department of Animal Science, North Carolina State University Raleigh, NC, USA; 3 Department of Plant Science, University of California-Davis, CA, USA

## 

Genetic merit can be considered the finite sum of thousands of allelic effects, each physically located at some place on the genome, whose transmission can be traced through molecular markers. Traditionally, best linear unbiased prediction (BLUP) of breeding values relies on average additive genetic covariances (the numerator relationship matrix **A**) derived from pedigrees to utilize information from relatives. For example, all pairs of full-sib offspring of a cross are assumed to share 50% of alleles in common. Such assumptions ignore variation in Mendelian segregation of alleles among progeny within family. With advances in marker genotyping technology and reduction in genotyping cost, it is now feasible to estimate genetic covariances from markers.

Linear mixed models that utilize realized genomic relationship matrices could predict genomic estimated breeding values (GEBV) more accurately than those that use expected average genetic covariances derived from pedigrees. Dense markers can be used to trace identity by descent probabilities at each locus, and those probabilities used to construct an incidence matrix. The incidence matrix is used to estimate the genomic relationship matrix (**G**), which is used in place of the **A** matrix in solving the mixed model equations. This may allow more accurate estimation of individual breeding values than the traditional model based on average genetic covariances.

We estimated realized genetic covariances between cloned progeny of a *P. taeda* population. There were 165 cloned progeny derived from nine full-sib families. The realized genomic relationships were based on a set of 3,461 biallelic SNP markers. We used the following linear mixed model **y** = **Xb** + **Zu** + **e** to estimate GEBV. In the model **X** and **Z** are incidence matrices, **b** is the vector of fixed mean, **u** is the vector of additive genetic effects that correspond to allele substitution effects for each marker with Var (**u**) = **I**σ^2^_m_; where σ^2^_m_ is the marker variance and **I** is the identity matrix. The term **e** is the vector of residuals. The dimension of **Z** is the number of individuals (n) by the number of loci (m). The regression method used to construct our **G** matrix did not require allele frequencies; instead, the inverse of the **G** matrix was generated by regressing **ZZ’** as a dependent variable on the **A** matrix as the independent variable. Therefore, the expected value of **G** is **A** plus a constant matrix.

Different cross-validation methods were used to test performance of the** G** matrix. Clones were divided into a training group with both marker and phenotypic information and a validation group for which only marker genotypes were used. In one scenario ~90% of the clones (148) were sampled for training, either randomly selecting within each of the nine families or at random without family consideration. The remaining ~10% were used for validation (17 clones). In the second scenario, ~50% of clones (84) were sampled either within family or randomly from the whole population for training, and the remaining ~50% were used for validation (81 clones). The model parameters estimated in the training set were used to predict GEBV in the validation set. For each scenario, six independent samplings were carried out. The mean correlation between the GEBV based on **G**-BLUP and breeding values based on **A**-BLUP were determined for each scenario, along with the accuracy of the BLUP predictions for both **G** and **A** based models. The mean correlation varied from 0.37 to 0.61 across the four validation methods. The accuracies of the predictions for any validation scenario were always higher for **G**-BLUP (range of 0.65 to 0.75) than **A**-BLUP (0.60 to 0.62), which is related to the smaller standard error of the predicted **G**-BLUP for the validation clones (17 or 81) under the different scenarios.

Estimating realized genetic covariances based on the genotypes of biallelic markers and incorporating those estimates into G-BLUP helps to more accurately characterize Mendelian segregation of alleles, and could allow more accurate selection within family. Such a method would increase genetic gains in forest tree breeding. The major impact would be on reducing the need for expensive field testing, but it may also be possible to shorten the breeding cycle and thus increase genetic gain per unit time and cost. The impact of genomic selection applications in forest tree breeding may be greater than for other crop or animal species, because of the biology of trees and their much longer breeding cycles.

